# Exercise reduced the formation of new adipocytes in the adipose tissue of mice *in vivo*

**DOI:** 10.1371/journal.pone.0244804

**Published:** 2021-01-20

**Authors:** Timothy D. Allerton, Jonathan J. Savoie, Mark D. Fitch, Marc K. Hellerstein, Jacqueline M. Stephens, Ursula White

**Affiliations:** 1 Pennington Biomedical Research Center, Louisiana State University System, Baton Rouge, LA, United States of America; 2 University of California at Berkeley, Berkeley, CA, United States of America; West Virginia University, UNITED STATES

## Abstract

Exercise has beneficial effects on metabolism and health. Although the skeletal muscle has been a primary focus, exercise also mediates robust adaptations in white adipose tissue. To determine if exercise affects *in vivo* adipocyte formation, fifty-two, sixteen-week-old C57BL/6J mice were allowed access to unlocked running wheels [Exercise (EX) group; n = 13 males, n = 13 females] or to locked wheels [Sedentary (SED) group; n = 13 males, n = 13 females] for 4-weeks. *In vivo* adipocyte formation was assessed by the incorporation of deuterium (^2^H) into the DNA of newly formed adipocytes in the inguinal and gonadal adipose depots. A two-way ANOVA revealed that exercise significantly decreased new adipocyte formation in the adipose tissue of mice in the EX group relative to the SED group (activity effect; P = 0.02). This reduction was observed in male and female mice (activity effect; P = 0.03). Independent analysis of the depots showed a significant reduction in adipocyte formation in the inguinal (P = 0.05) but not in the gonadal (P = 0.18) of the EX group. We report for the first time that exercise significantly reduced *in vivo* adipocyte formation in the adipose tissue of EX mice using a physiologic metabolic ^2^H_2_O-labeling protocol.

## Introduction

Exercise has favorable effects on metabolic health, including improvements in glucose homeostasis and cardiovascular outcomes. Skeletal muscle has been the primary focus in exercise studies, as beneficial effects on insulin sensitivity and mitochondrial biogenesis are well-characterized [1, 2]. However, exercise also mediates robust adaptations in other tissues, including white adipose tissue (AT). A seminal study reported that the transplantation of subcutaneous AT from exercise-trained mice to sedentary, insulin resistant mice resulted in improved glucose homeostasis in the recipient mice [3], demonstrating that exercise-induced changes in AT can have favorable effects on metabolism.

White AT is a dynamic organ that is characterized by changes in adipocyte volume, as well as continual adipocyte formation (i.e. hyperplasia; adipogenesis) and death, which all regulate AT mass. Studies in rodents [4] and humans [5, 6] have highlighted the significant link between adipocyte turnover (e.g. formation and death) and health outcomes, such as obesity and related disorders. Although important adaptations of AT, such as changes in mitochondrial activity [7, 8], morphology [9–11] and endocrine function [10, 12], have been reported, there is a paucity of data on the effects of exercise on *in vivo* adipogenesis and adipocyte turnover.

In this study, we assessed *in vivo* adipogenesis using a metabolic labeling protocol [13] that incorporates deuterium (^2^H), administered as ^2^H_2_O, into the DNA of adipocytes in the white AT depots of mice that were sedentary (SED) or exercising via voluntary wheel running (EX) for 4-weeks. Using this practical ^2^H_2_O approach, we report for the first time that exercise significantly reduced new adipocyte formation (e.g. adipogenesis) in the AT of EX mice. These findings suggest that decreased adipocyte formation may be an important exercise-induced mechanism of AT remodeling.

## Materials and methods

### Animals

Fifty-two, twelve-week-old male (n = 26) and female (n = 26) C57BL/6J mice were purchased (The Jackson Laboratory, Bar Harbor, ME) and housed at room temperature in the PBRC comparative biology core. Mice were multi-housed (4 to cage) and fed a standard rodent chow diet for 4 weeks. After the 4 weeks (16 weeks of maturation), body composition was measured by nuclear magnetic resonance (NMR) as previously described [14], and mice were then single housed in cages with locked running wheels for an adaptation period of one week.

### ^2^H_2_O labeling, body ^2^H_2_O enrichments, and exercise intervention

At the start of the study, all mice were given a bolus injection of ^2^H_2_O (35 ml/kg body weight 0.9% NaCl in 100% ^2^H_2_O) (Cambridge Isotope Laboratories, Andover, MA) to bring the ^2^H_2_O content of the body water up to approximately 5% [13]. After the injection, mice were provided *ad libitum* access to 8% ^2^H_2_O drinking water and low-fat diet (10% fat) during the 4-week intervention period to maintain ^2^H_2_O content in the body water.

Mice were then randomly assigned to the SED and EX groups, with an equal number of males and females in each group. Half of the mice were allowed access to unlocked running wheels (EX group; n = 13 males, n = 13 females) or to wheels that remained locked to serve as sedentary controls (SED group; n = 13 males, n = 13 females) for 4-weeks. Food intake (grams) and body weight (grams) were measured weekly. Wheel running data was collected weekly for total wheel revolutions and converted into kilometers. To evaluate the changes in body composition (e.g. total fat mass (g) during the 4-week intervention period, NMR (Minispec LF50 TD, Bruker) was performed again post-intervention, as previously described [14]. The Δ adipose tissue mass was calculated by subtracting the final total fat mass values at the end of the study from the value prior to the intervention. Mice were euthanized by isoflurane administration with cardiac puncture. Pennington Biomedical Research Center's Institutional Animal Care and Use Committee (IACUC) approved the protocol for animal care and use (#1008).

The enrichment of ^2^H_2_O in body water (blood serum) in the mice was measured by isotope ratio mass spectrometry. Serum samples were distilled, and the evaporated water (distillate) was collected. The isotope enrichment in the distillate was directly analyzed with an H-Device attached to a Delta V Advantage Mass Spectrometer. ^2^H_2_O enrichments were calculated by comparison to standard curves generated by mixing 100% ^2^H_2_O with natural abundance ^2^H_2_O in known proportions.

### Adipose tissue analysis

Inguinal (iWAT) and gonadal (gWAT) adipose tissue depots were carefully dissected and weighed prior to analysis.

#### Isolation of adipocytes

Adipose tissues were treated with a HEPES/type 1 collagenase solution for ~1hr at 37°C to isolate adipocytes and the stromal-vascular fraction (SVF). The adipocytes were purified by incubation with a cocktail of antibodies against markers of endothelial cells (CD31- eBioscience), hematopoietic cells (CD45- BioLegend), and mesenchymal stem cells (CD34- eBioscience) for 15min at room temperature. Cells attached to these antibodies were removed by magnetic nanoparticles (EasySep), while immuno-purified adipocytes were retained. Adipocytes were flash frozen in liquid N_2_ and stored at -80°C until DNA extraction.

#### Collection of bone marrow

After removing the femurs, the ends were cut to allow access to the bone marrow. Each femur was placed in a 1.5 ml tube and centrifuged at 14,000 RPM for 1 minute to acquire a marrow pellet for deoxyribose analysis from rapidly turning over bone marrow cells [15].

#### Deozyribose analysis

DNA was isolated from the adipocytes and bone marrow using DNEasy microDNA extraction kits (QIAGEN). After isolation, samples were hydrolyzed overnight at 37°C in an enzyme cocktail containing S1 nuclease and phosphatase enzymes. Following hydrolysis, 100 μL of pentafluorobenzyl hydroxylamine hydrochloride (1 mg/mL) and 75 μL of glacial acetic acid were added to each sample followed by incubation at 100°C for 30 minutes. After cooling, 2 mL of acetic anhydride and 100 μL of 1-methylimidazole were added followed by 100°C heat block for 5 minutes, then allowed to cool. Afterwards, 3 mL of HyClone water was added to each sample, vortexed, and let sit for 10 minutes. Then, 2 mL dichloromethane were added, and samples were vortexed vigorously for 15 seconds. After centrifugation at 1500 RPM for five minutes, the bottom dichloromethane layer was transferred into a clean 16 x 100 test tube, evaporated to dryness under nitrogen for 30 minutes, followed by a 30 minute dry in a speed vacuum (Labconco, MO, USA) at room temperature.

#### Gas chromatographic and mass spectrometric analyses

Once dry, the samples were resuspended in 150 uL of ethyl acetate for GCMS analysis using Agilent 6890N Gas Chromatography system with a DB-17MS capillary column (30m, 0.25mm, 0.25 μm, J&W Scientific) and a 5975B inert XL EI/CI MSD (Agilent, Santa Clara, CA, USA). Samples were analyzed via negative chemical ionization using helium as the carrier gas and methane as the reagent gas. Deoxyribose enrichment was measured using selective ion monitoring for molecular ions 435, 436, and 437 m/z corresponding to M0, M+1, and M+2. The samples were injected using pulsed splitless with a constant flow of 1 mL/min and a run time of 16 minutes. Ion abundances were analyzed using the Quantitative Mass Hunter Workstation (Agilent, Santa Clara, CA, USA). Baseline (unenriched) DNA standards were measured concurrently to correct for abundance sensitivity.

#### Calculation of the fraction of new adipocytes

The enrichment of the M1 ion above natural abundance in the adipocyte samples of interest (%EM1 enrichment in adipocytes) was determined by subtracting the %M1 in unenriched DNA standard from the %M1 in the sample, as determined in each case by %M1 = M1 ion/(M0+M1+M2 ions). The theoretical maximum enrichment, %EM1, in adipose cells was calculated using MIDA equations based on the body ^2^H_2_O exposure (serum) [15]. The fraction of new adipocytes (%), or the formation of new adipocytes, as reported in the Results section, is calculated by the following:

$$Fraction\;of\;new\;adipocytes\;\left(\%\right)=\frac{\%EM1\;enrichment\;in\;adipocytes}{Theoretical\;maximum\;\%EM1\;enrichment}X\;100$$

Bone marrow from each mouse was collected and analyzed to represent a (near) completely turned-over cell pulation. This measurement, used as a reference marker of ^2^H_2_O exposure, serves to confirm calculations using the theoretical maximum enrichment based on the body ^2^H_2_O exposure measured post-mortem.

### Statistical analysis

Independent and paired *t*-test and two-way ANOVA were utilized to analyze data where appropriate (Prism, Graphpad, version 8 and JMP Pro version 14.2, SAS Institute Inc.). Multiple pairwise comparisons were made by Holm-Sidak’s test. Main effects for activity were based on activity (exercise vs. sedentary), adipose tissue depot (iWAT vs. gWAT), and sex (males vs. females). To measure the fraction of new adipocytes, or the formation of new adipocytes, adipose tissue samples were pooled (2–3 mice per sample). Statistical significance was declared at P = 0.05. All data are reported as mean ±SEM.

## Results

**Fig 1:**
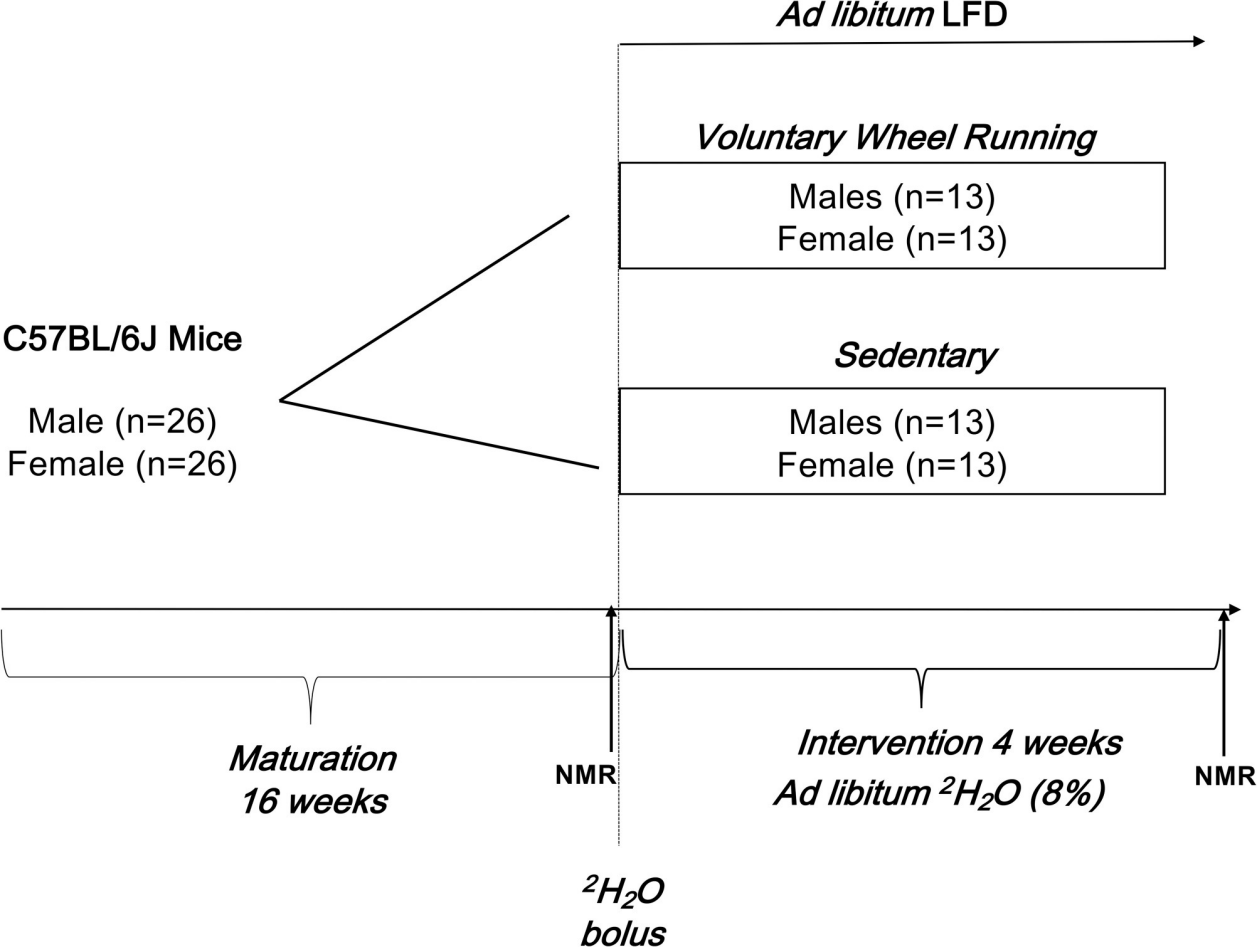
Study design schematic. *In vivo* adipocyte formation was examined in fifty-two male (n = 26) and female (n = 26) C57BL/6J mice. Twelve-week old mice were fed a standard chow diet until 16 weeks of age. Mice were then switch to single housed cages with locked running wheels. After an adaptation period of one week, all were given a bolus injection of ^2^H_2_O (35 ml/kg body weight 0.9% NaCl in 100% ^2^H_2_O) and provided 8% ^2^H_2_O drinking water and low-fat diet (10% fat). Body composition was measured by NMR at week 16 and week 20. Half of the mice were then allowed access to unlocked running wheels (EX group; n = 13 males, n = 13 females) or to wheels that remained locked to serve as sedentary controls (SED group; n = 13 males, n = 13 females) for 4-weeks.

**Fig 2:**
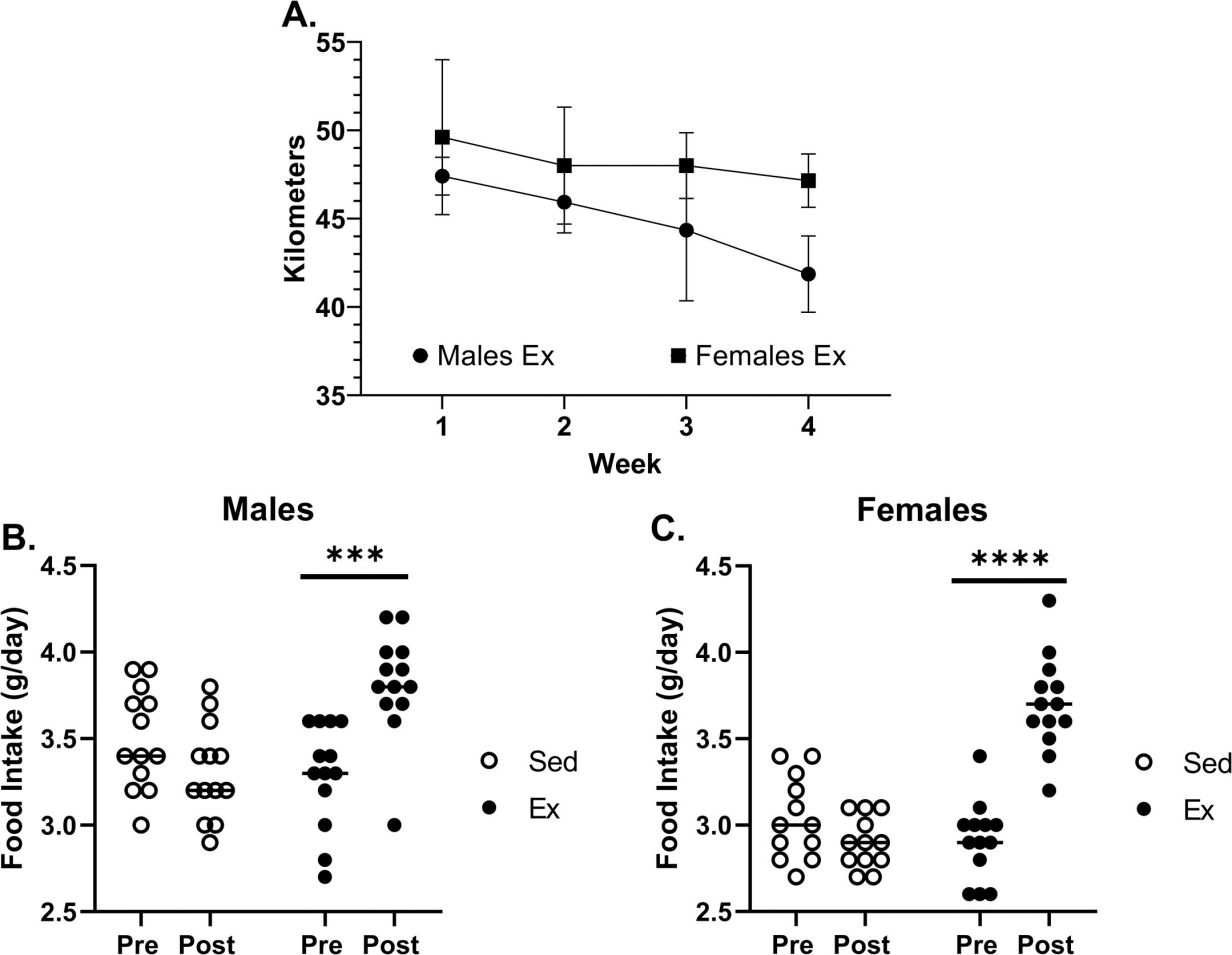
Effects of exercise on wheel running kilometers and food intake in male and female mice. Singly housed male and female mice were monitored for 4-weeks following a bolus injection of ^2^H_2_O to achieve ~5% body water enrichment. Mice were provided ad libitum access to 8% ^2^H_2_O drinking water (to maintain body water enrichment) and switched to low fat diet at start of the intervention. During the 4-week intervention period, half of the mice were allowed access to an unlocked running wheel (EX group; n = 13 males, n = 13 females), while the other mice had access to wheels that remained locked to serve as sedentary controls (SED group; n = 13 males, n = 13 females). Wheel running (2A) and food intake (2B-C) were measured weekly in male (n = 13) and female (n = 13) mice. g, grams; VWR, voluntary wheel running; EX, Exercise group; SED, Sedentary group. All data are presented as Mean ± SEM.

**Fig 3:**
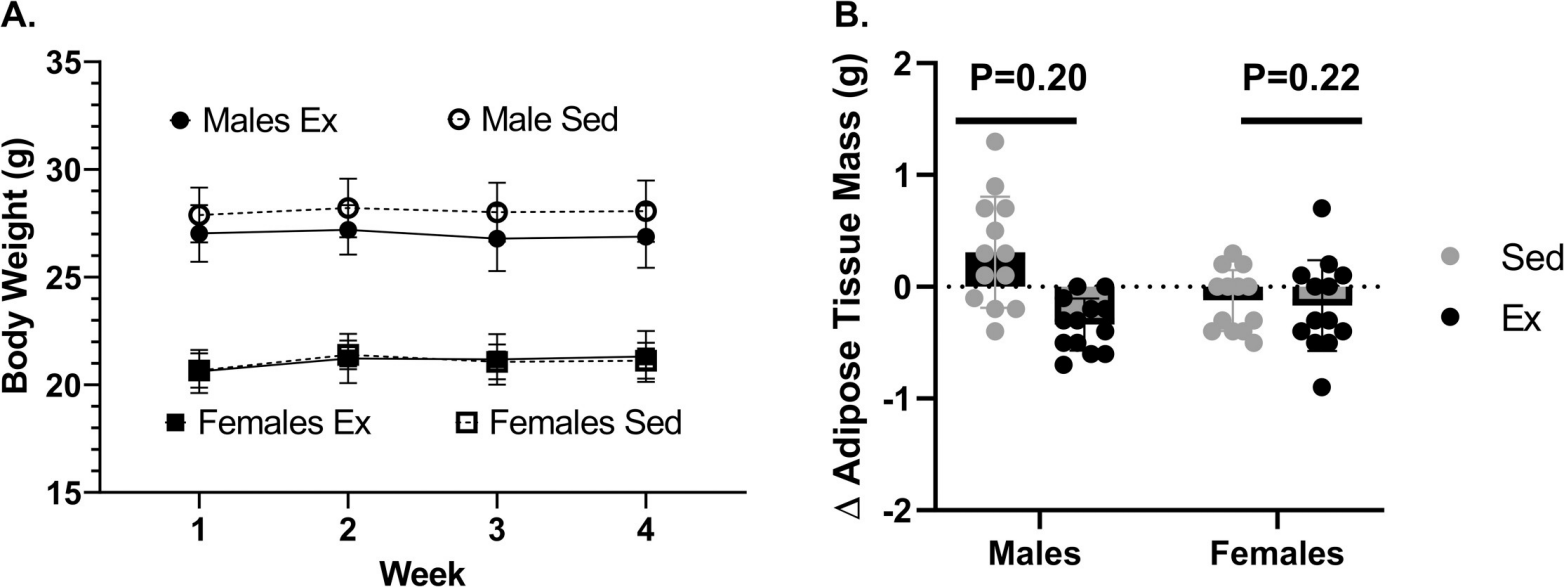
Changes in body weight and fat mass during the 4-week intervention period. (A) Body weight was measured weekly in the mice during the study period. (B) NMR was performed to measure adipose tissue mass prior to the exercise period (baseline) and post-intervention. The change (Δ) in adipose tissue mass during the intervention period was calculated as ‘AT mass post-intervention–AT mass baseline’. n = 13/group. g, grams. All data are presented as Mean ± SEM.

**Fig 1** provides a schematic of the study design. The average body ^2^H_2_O enrichment, as measured in the blood serum, was 4.2 ± 0.3% in the SED group and 4.1 ± 0.5% in the EX group, with no significant difference between the groups (p = 0.53). Within the exercising (EX) group, there were no significant differences in total voluntary wheel running (VWR) between male (179 ± 8.0 km) and female (196 ± 9.9 km) mice (P = 0.06) **(Fig 2A)**. When comparing pre- versus post-exercise food intake (g/day), both male (3.28 ± 3.81 g/day; P = 0.0002) and female (2.91 ± 3.68 g/day; P<0.0001) mice in the EX group significantly increased food consumption (**Fig 2B and 2C)**. There were no changes in food intake for mice in the SED group (males; P = 0.18 & females; P = 0.62). Body weight measures over the 4 weeks did not differ between the EX and SED groups within sexes (males, P = 0.32; females, P = 0.88), but as expected was different between sexes (P<0.0001) regardless of activity (EX vs. SED) **(Fig 3A)**. No significant differences in final body weight were observed between SED and EX groups for male (27.9 ± 1.8 vs 26.8 ± 1.4 g; P = 0.07) or female mice (21.1 ±0.8 vs. 21.3 ± 1.3 g; P = 0.63). There were no significant differences for the Δ AT mass between male (P = 0.20) or female (P = 0.22) SED and EX mice **(Fig 3B)**.

**Fig 4:**
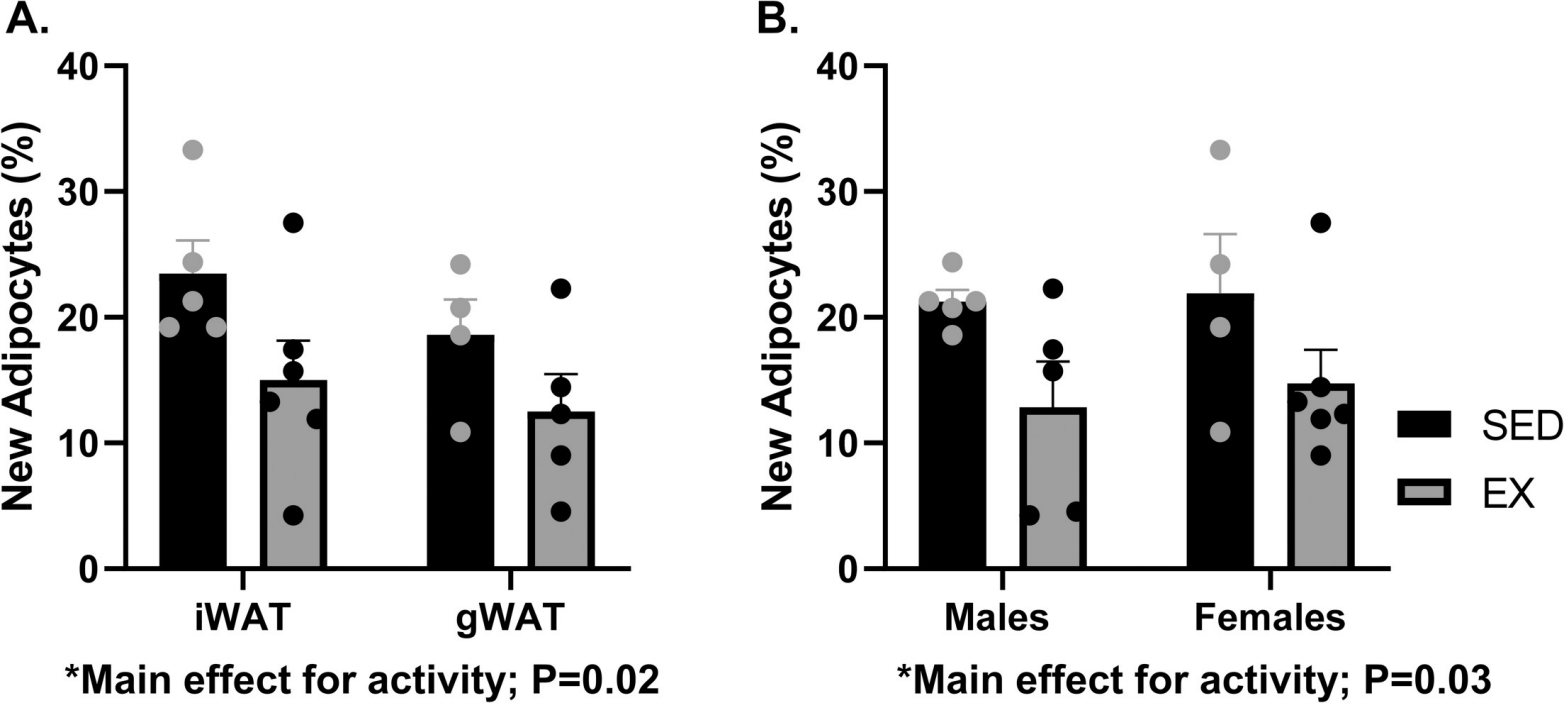
New adipocyte formation is reduced in exercising mice. New adipocyte formation was compared between SED and EX mice in the iWAT and gWAT (main effect for activity; p = 0.02) and in males and females (main effect for activity; p = 0.03). Additional independent pairwise analyses by depot (4A) and sex (4B) were conducted comparing new adipocyte formation in SED versus EX mice. n = 13/group. iWAT, inguinal white adipose tissue; gWAT, gonadal white adipose tissue; EX, exercise; SED, Sedentary. All data are presented as Mean ± SEM.

A two-way ANOVA of activity versus depot revealed that exercise significantly decreased the fraction of new adipocytes, or adipocyte formation, in the AT of mice in the EX relative to the SED group (activity effect; P = 0.02). Pairwise analysis of the inguinal (iWAT) and gonadal (gWAT) depots (male and female) revealed a significant reduction in adipocyte formation in the iWAT (SED, 24.5 ± 6.2%; EX, 15.02 ± 7.6%; P = 0.05) but not in the gWAT (SED, 18.6 ± 5.6%; EX, 12.5 ± 6.6%; P = 0.18) in the EX as compared to the SED group **(Fig 4A)**. There was no significant difference in the formation of adipocytes between iWAT and gWAT for EX (P = 0.54) or SED (P = 0.23) mice.

A two-way ANOVA of activity versus sex revealed that exercise significantly reduced new adipocyte formation in male and female mice of the EX group as compared to the SED group (activity effect; P = 0.03). Pairwise analysis of sex (iWAT and gWAT depots) showed that exercise tended to decrease new adipocyte formation in males (SED, 21.2 ± 2.4%; EX, 12.8 ± 8.0%; P = 0.08) and females (SED, 21.9 ± 9.3%; EX, 14.7 ± 6.5%; P = 0.13) of the EX group as compared to the SED group, though not statistically significant **(Fig 4B)**. There was no statistical difference in new adipocyte formation between male and female mice for the EX (P = 0.67) or the SED groups (P = 0.89).

## Discussion

Adipocytes are constantly formed and replaced in rodents [4, 13, 16] and humans [17–19]. We evaluated the effects of exercise on *in vivo* adipogenesis in mice using a practical ^2^H_2_O metabolic labeling approach and report for the first time using this methodology that voluntary wheel running resulted in reduced adipocyte formation. Prior studies have reported exercise effects on the AT in the context of weight-loss [9, 20]; however, it is plausible that the observed effects could be partially attributed to the weight loss as opposed to the exercise. Moreover, the health benefits of exercise can occur without significant weight-loss [21]. Our observation of no significant differences in changes in body weight or the loss of AT mass between sedentary and exercising mice is a strength of this study.

Many studies have shown that AT remodeling during exercise is associated with metabolic improvements [2, 5, 8, 22], including reduced adipocyte size and triacylglycerol content [9–11, 23]. Mitochondrial enzyme activity is also increased in the AT of exercise-trained rodents [7, 8, 24, 25] and humans [26, 27], which may be associated with increased fat oxidation. Exercise-induced changes in AT has also been associated with improvements in glucose metabolism and protection against inflammation in rodents [24, 28, 29]. Histological analysis of iWAT from exercised mice revealed the presence of multilocular cells with increased vascularization [30], supporting studies that demonstrate “beiging” of white AT [3, 31].

Adipocyte turnover is an essential function that affects systemic metabolism [6, 32, 33]; yet, the impact of exercise on this AT feature has not been extensively explored. Only one other study reported that exercise could attenuate the proliferation and differentiation potential of adipose cells in transgenic male mice using bromodeoxyuridine (BrdU) and green fluorescent protein (GFP) to label cells expressing PPARγ [20]. Our present study utilized a distinct ^2^H_2_O-labeling approach that has been validated to provide physiological, quantitative measures of *in vivo* adipocyte formation in rodents and humans. Though informative in rodents, the use of BrdU and GFP labelling, as used in Zeve et al. [20], involve several limitations, including toxicity and biochemical complications and, therefore, are not applicable for use in humans. Moreover, it is well-known that PPARγ expression is not adipocyte specific and present in other cells, including macrophages. By utilizing a practical *in vivo* assessment of adipogenesis, we demonstrate that exercise attenuates new adipocyte formation in a sex-independent manner. Further studies are needed to determine how reduced adipocyte formation contributes to exercise-induced improvements in metabolism, as this was not investigated in our current experiment. Nevertheless, our findings suggest that decreased adipocyte formation may be an important exercise-induced mechanism of AT remodeling and provide the foundation for further sophisticated studies in rodents and humans with obesity and related disorders using this *in vivo* approach.

At a steady state in AT mass, adipocyte turnover represents adipocyte formation and death. Our findings are consistent with recent studies reporting that lower adipocyte formation, or turnover, in humans is observed in conditions of more favorable metabolic health [33, 34]- results that are contrary to the AT expandability hypothesis [5, 6]. It could be speculated that increased adipogenesis and turnover reflect adipocyte fragility and death, which can lead to recruitment of macrophages, unfavorable remodeling and inflammation [35].

This study was conducted in lean, healthy mice. Hence, we do not report data on exercise-induced improvements in metabolism, and our observations are not generalizable to mice with metabolic dysfunction. Further studies in an obesogenic environment are necessary to understand the effects of exercise on adipocyte turnover during periods of energy surplus. Our findings report exercise-mediated effects on adipocyte formation, independent of a significant loss in body weight and fat mass during the 4-week intervention period. Nevertheless, the long-term effects of exercise on adipocyte turnover and how this impacts prospective energy balance, body weight, and adipose tissue mass cannot be determined from this study and require interventions that are longer in duration (than 4 weeks). Another limitation of the study is that we did not measure changes in triglyceride kinetics (via the ^2^H_2_O method), adipocyte morphology (fat cell size), or the expression of genes related to adipocyte metabolism in this initial observation, due to a limited amount of available AT. Future studies will include a more comprehensive assessment of other cell populations and facets of adipose tissue remodeling that occur in response to exercise.

## Conclusions

We demonstrate the feasibility to measure *in vivo* adipogenesis in the AT of exercising and sedentary mice using a metabolic ^2^H_2_O-labeling approach. Our observations provide evidence of a reduction in the formation of new adipocytes in response to exercise. Future studies are necessary to further elucidate the role of adipose turnover in mediating the effects of exercise on metabolism and health.
